# Human color constancy in cast shadows

**DOI:** 10.1177/20416695251349737

**Published:** 2025-07-15

**Authors:** Takuma Morimoto, Masayuki Sato, Shoji Sunaga, Keiji Uchikawa

**Affiliations:** 1 University of Oxford, UK; 2 Physics Center of Minho and Porto Universities (CF-UM-UP), Braga, Portugal; 3 12876 University of Kitakyushu, Japan; 4 Kyushu University, Fukuoka, Japan; 5 Human Media Research Center, 12856 Kanagawa Institute of Technology, Atsugi, Japan

**Keywords:** color constancy, cast shadow, sunlight, skylight, achromatic setting

## Abstract

Illumination conditions inside and outside cast shadows typically differ significantly in both intensity and in chromaticity. However, our daily experiences suggest that we generally have no difficulty in stably perceiving surface color in cast shadows. In this study, two experiments were conducted to measure the extent to which color constancy holds within cast shadows. We constructed a scene with colored hexagons illuminated by two projectors simulating “sunlight” and “skylight.” Part of the scene included a cast shadow, illuminated only by the skylight, where a subjective white point was measured. We also created a condition in which a cast shadow was not perceived as a shadow. Results showed that color constancy generally holds well in shadows, and the color of skylight had varying effects depending on observers. Perceiving a cast shadow as a shadow had no effect. Overall, these findings are consistent with our daily experiences, in which we stably judge objects’ color even within cast shadows.

## How to cite this article

Morimoto, T., Sato, M., Sunaga, S., and Uchikawa, K. (2025) Human color constancy in cast shadows. *i-Perception, 16*(4), 1–17. https://doi.org/10.1177/20416695251349737

## Introduction

Our ability to judge the surface color of an object remains stable despite large variations in lighting environments, a phenomenon known as color constancy. The extent to which color constancy holds has been measured under diverse experimental conditions, with various strategies proposed to discount the influence of illumination (Foster, 2011). However, a key limitation of these studies is that they often consider scenes lit by a single light source (e.g., Maloney & Wandell, 1986; Morimoto et al., 2016, 2021a), and there are only a few behavioral studies investigating the mechanisms of color constancy in scenes with multiple illuminations (Boyaci et al., 2004; Doerschner et al., 2004, 2007; Lee & Smithson, 2012; Smithson & Zaidi, 2004; Yang & Shevell, 2003).

**Figure 1.**
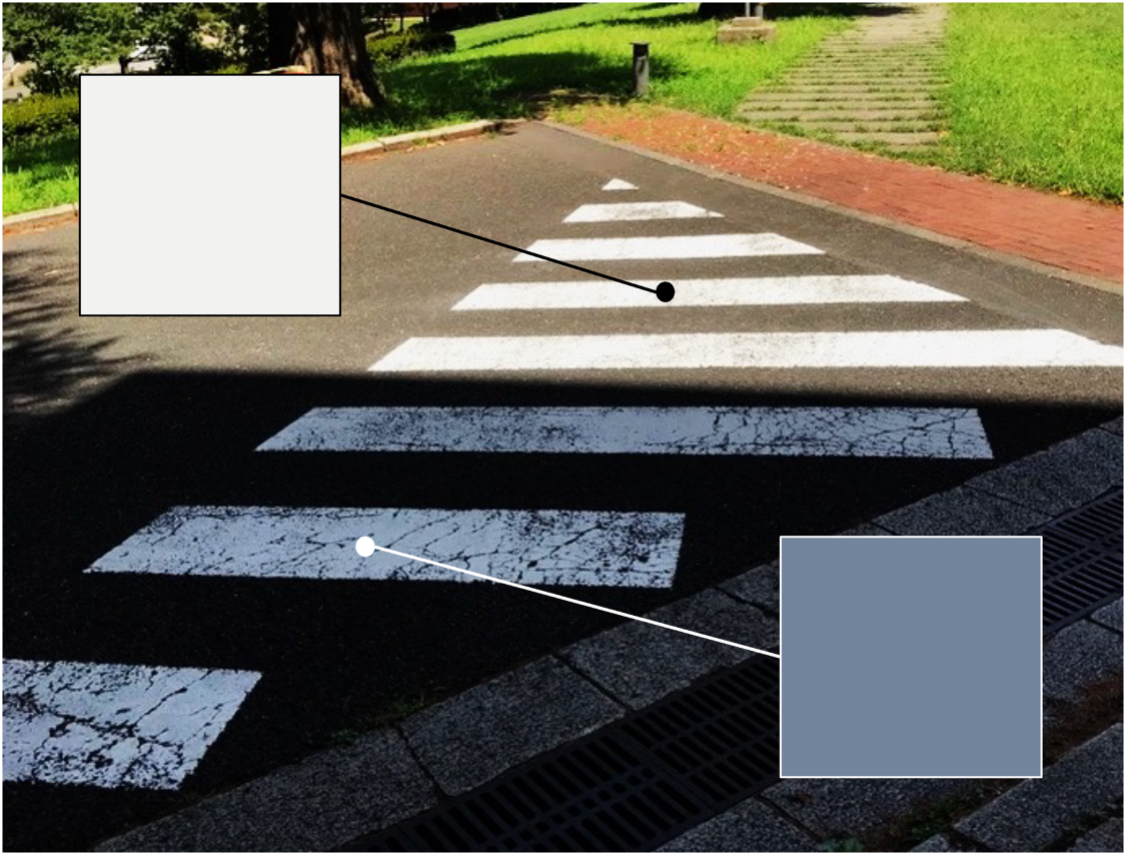
The white pedestrian crossings appear white to our eyes, whether in shadow or sunlight. However, as indicated by the two squares, the pixel colors inside and outside the shadow differ drastically. Photograph taken by an author at Ookayama Campus of the Institute of Science Tokyo.

Yet, such multi-illuminant conditions are not rare in natural environments. For example, sunny outdoor scenes typically contain two major illuminants, a sunlight and a skylight (Preetham et al., 1999; Tian et al., 2016; Wilkie et al., 2021). Color constancy in a multi-illuminant environment is inherently more challenging for our visual system than in a single-illuminant environment because the influence of illuminant needs to be inferred differently for different spatial locations. One further complication is that the occlusion of one light generates a cast shadow under which the influence of the illuminants from other directions becomes dominant, creating a complex spatial lighting variation. Figure 1 shows a photograph taken by an author at the Ookayama Campus of the Institute of Science Tokyo in Japan. Readers can presumably recognize that the pedestrian crossings appear white regardless of whether they are in sunlight (upper part) or shadow (lower part). However, as depicted by squares in the photograph, the pixel color for the shadowed region is dark blue due to skylight reflection, which contrast sharply with the pixel color of the sunlit region, even though both areas are painted with the same material. This example demonstrates that our visual system can stably judge surface colors under spatial illuminant variations. While lightness, brightness, and shape perception in shadows have been relatively well studied (Adelson, 1999; Knill et al., 1997; MacLeod, 1940; Mamassian et al., 1998; Soranzo & Agostini, 2004) there is little empirical research available on color constancy in shadows (Newhall et al., 1958), despite the concept being documented several hundred years ago (Mollon, 2006; Thompson, 1794).

To illustrate the computational challenge our visual system faces in shadows, we consider their physical characteristics. While measuring daylight spectra has been an active area in color vision studies (e.g., Hernández-Andrés et al., 2001; Judd et al., 1964), only a few studies have specifically measured the light reaching cast shadows (Yu et al., 2023; Yu & Pont, 2021). We also previously measured the spectral composition of light reaching a cast shadow (i.e., skylight) in an outdoor environment, as well as light reaching areas under the sun (i.e., direct sunlight plus skylight) from dusk till dawn (Morimoto et al., 2022). As expected, the measurements revealed significant spectral differences between the two regions. On sunny days, skylight is the dominant light source reaching shadowed areas, with spectra high in energy around the short wavelength region. In contrast, nonshadowed areas receive much brighter sunlight, and their chromaticity is located around the white or slightly yellowish region in a color space. This complexity created by shadows has naturally attracted researchers in the field of computer vision, leading to the development of a wide range of methods for automatic detection and removal of shadows over the decades (Cun et al., 2020; Le & Samaras, 2022; Liu et al., 2021; Lu & Drew, 2005; Qu et al., 2017; Wang et al., 2017); as well as more general algorithms and machine learning models to estimate multiple illuminants in a scene (Das et al., 2021; Gijsenij et al., 2012; Qu et al., 2015; Wang et al., 2022). It is thus intriguing to consider how humans effortlessly detect and discount the influence of a shadow.

The purpose of this study was to measure how effectively we can perceptually discount the influence of illumination within cast shadows and to identify factors that might affect the degree of constancy. We conducted two psychophysical experiments. In both experiments, we constructed a real scene lit by two independent light sources simulating “sunlight” and “skylight,” provided by two liquid crystal projectors to mimic outdoor environments. The scene included a cast shadow where an achromatic setting was measured. Overall, we addressed three key questions in this study.

1. How well does color constancy function within cast shadows? (Experiment 1).
2. Does the color of the skylight affect the degree of color constancy? (Experiment 1).
3. Does outlining the edge of the cast shadow impact the degree of color constancy? (Experiment 2).

Regarding the first point, due to the lack of recent empirical assessment, we believed that formally measuring color constancy in shadows should be a starting point. The second point aimed to test the idea that human observers use statistical chromatic regularities in outdoor scenes, where shadowed areas tend to be illuminated by blue skylights on sunny days, neutral lights on cloudy days, and yellow or orange lights during sunset. This was directly inspired by a past empirical measurement showing these regularities (Morimoto et al., 2022), but this is of wider interest to the field, especially since in color constancy literature there has been a similar argument regarding the daylight prior, though its existence remains inconclusive (Delahunt & Brainard, 2004; Morimoto et al., 2021b; Pearce et al., 2014; Weiss et al., 2017). The third point is based on our hypothesis that perceiving a cast shadow as a shadow is necessary to judge that the shadowed region is illuminated differently from other regions. We achieved the condition by outlining the edge of the shadow (penumbra) with black surfaces, inspired by Hering's outlined shadow (Hering, 1964), where a cast shadow appears as a grey spot when a broad black line covers the blurred contours of the shadow.

## General Method

### Experimental Apparatus

For both experiments, we set up a real scene using two independent liquid crystal projectors (HI-04, 1920 × 1080 pixels, 3600 lumens, DR. J Professional, Kent, Germany) to simulate “sunlight” and “skylight.” The projectors were not visible from the observers’ viewpoints. There were two types of scenes.

**Figure 2.**
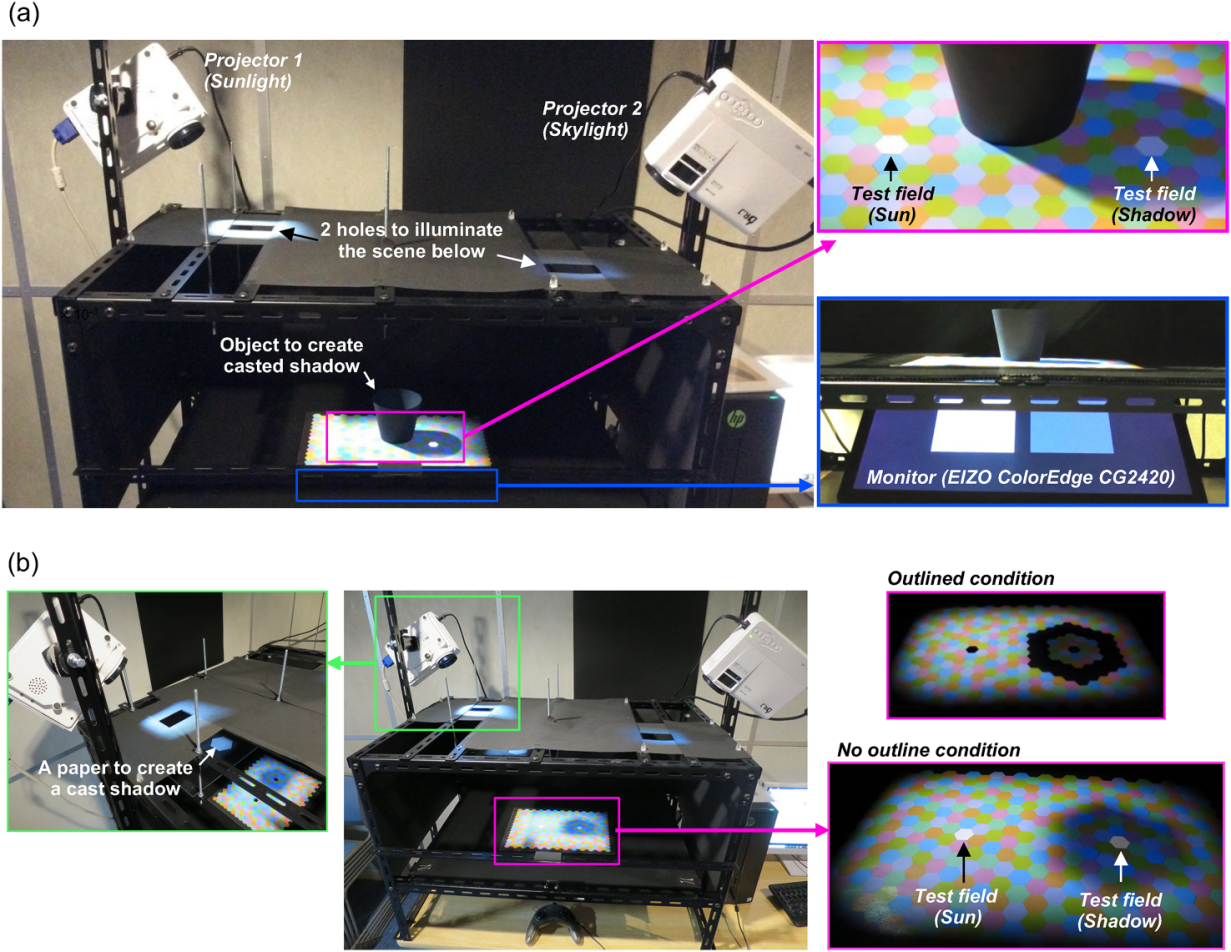
(a) Experimental setup for Experiment 1. Lights from two projectors illuminate the scene, with a central black object creating a cast shadow. (b) Setup for Experiment 2. The center black object was removed, and a white paper was placed in the optical path between the left projector and the scene.

Figure 2a shows the setup used in Experiment 1. The scene consisted of a sheet of colored hexagons arranged without spatial gaps. A central cup-shaped black object was placed to cast a shadow over the right part of the sheet. The sheet contained two test fields (holes) within the magenta square. Below the sheet, we placed an experimental monitor (ColorEdge CG2420, 24.1 inches, 1920 × 1200 pixels, 60 Hz, EIZO, Ishikawa, Japan) shown within the blue square, which modulated the chromaticity and the luminance of the test fields. Each hexagon (including test fields) extended ∼2.5° horizontally, and the entire color sheet extended 46° by 22°, horizontally and vertically, respectively. From the observers’ viewpoint, the holes were perceived as surfaces, and they appeared to change color when the color of the monitor changed, as reported in previous studies (Morimoto et al., 2017; Uchikawa et al., 2001). In Experiment 1, both projectors were on in one condition, and only the right projector (skylight) was on in another condition. The physical properties of test surfaces and illuminants are described in the next subsection.

Figure 2b shows the setup used in Experiment 2. We removed the center black object from the scene, as the purpose of this experiment was to test whether color constancy holds even when a shadow is not recognized as a shadow (by outlining the shadow with black hexagons). If instead, the object from Figure 2a were present, observers might expect that there should be a cast shadow, and shadowed regions could still be perceived as shadows despite our manipulation. Thus, to create a cast shadow on the sheet, we placed a white paper (seen in the green square, labeled as “a paper to create a cast shadow”) that blocked the “sunlight” emitted from the left projector. The paper was not visible to observers. Otherwise the scene configuration was identical to Figure 2a. In Experiment 2, both left and right projectors were always on.

Using a spectroradiometer (CS-2000, Konica Minolta, Tokyo, Japan) with 401 spectral channels (380–780 nm, 1 nm step), we performed spectral calibration to map RGB values to cone excitations and gamma correction to linearize monitor and projector outputs. The calibration was conducted separately for the experimental monitor and each of the two LCD projectors. Stockman & Sharpe 2-degree cone fundamentals were used to compute cone excitations (Stockman et al., 1999; Stockman & Sharpe, 2000).

### Properties of Test Surfaces and Illuminants

**Figure 3.**
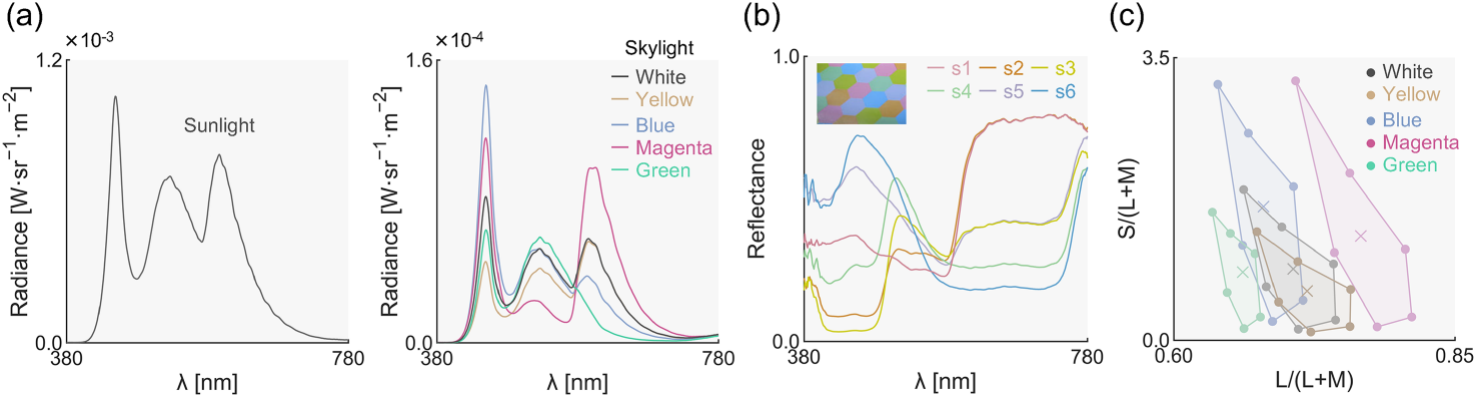
(a) Illuminant spectra of sunlight (left panel) and skylights (right panel). The *y*-axis range is different. (b) Spectral reflectances of six colored hexagons (s1–s6). (c) Filled circles show the chromaticities of the six colored hexagons under five skylights. Cross symbols depict the chromaticities of the five skylights.

The left and right projectors simulated sunlight and the skylight, respectively. The sunlight maintained a fixed spectral composition throughout the study (Figure 3a), with 2-degree *xy* chromaticity coordinates of *x* = 0.339 and *y* = 0.353. The skylight was set to one of five spectra shown in Figure 3b, chosen to represent typical (blue, yellow, and white) and non-typical variations (green and magenta). The 2-degree *xy* chromaticity for the skylights were blue (*x* = 0.259, *y* = 0.271), yellow (0.377, 0.379), white (0.334, 0.345), green (0.257, 0.395), and magenta (0.387, 0.258). The luminance of the sunlight was 41.9 cd/m^2^, while the skylights were substantially dimmer: 3.12, 3.14, 3.15, 3.15, and 3.00 cd/m^2^ for white, yellow, blue, magenta, and green, respectively. These spectral distributions and luminances were measured using a BaSO_4_ white calibration plate at the left test field for sunlight and the right test field for skylights. We also measured the spectral distribution at the left test field position when both sunlight and skylight were on. The measured spectrum was nearly identical to the plot in Figure 3a, with the influence of the skylight being negligible due to the intensity difference between the sunlight and skylights.

We printed a sheet with six colored hexagons using a color laser printer (SPC840, RICOH, Tokyo, Japan), as shown in the magenta rectangle in the upper-right corner of Figure 2a. We selected six colors to ensure that they are visually distinct when viewed under sunlight. The spectral reflectance of each color sample was measured using a spectrophotometer (CS-2000, Konica Minolta, Tokyo, Japan) from 400 to 780 nm at 1 nm intervals (Figure 3b). Figure 3c displays their MacLeod-Boynton chromaticities (MacLeod & Boynton, 1979) under five different skylight illuminants. The vertices represent the chromaticities of the six colors, and the colors of the data points correspond to the skylight color. In this figure, the *L*/(*L* + *M*) and *S*/(*L* + *M*) values for equal energy white are 0.7078 and 1, respectively. The average chromaticity across the six surfaces under the white skylight illuminant was 0.7062 and 0.8767 for *L*/(*L* + *M*) and *S*/(*L* + *M*), and 0.348 and 0.365 for 2-degree *x* and *y* coordinates.

### Observers

Three and 12 observers were recruited in Experiments 1 and 2, respectively. Two observers participated in both experiments, with no other overlaps between them. The group included one female, and the rest were male. The mean age and standard deviation of the observers were 57.0 and 8.72 in Experiment 1, and 28.3 and 14.8 in Experiment 2. All observers were screened for normal color vision using Ishihara pseudoisochromatic plates and had normal or corrected-to-normal visual acuity. Three observers in Experiment 1 and two observers in Experiment 2 were authors; one additional observer in Experiment 2 was aware of the purpose of the experiment. The remaining observers were naive to the experiment's purpose, with little or no experience in psychophysical experiments and no specialized knowledge of human color vision research. All observers completed all conditions in both experiments. The experimental procedures followed the guidelines and regulations of the University of Kitakyushu's ethics committee and adhered to the Helsinki Declaration.

### Task and Procedure

We employed the method of achromatic adjustment in all experiments (Brainard, 1998). While the original method involved adjusting chromaticity only, our experiments required observers to adjust both the luminance and chromaticity of a test field until it appeared as a full-white surface under a test illuminant (the “paper match” criterion Arend & Reeves, 1986). Here, “full-white surface” refers to the perceptually lightest white that is just below the luminance level where the surface appears to emit light. Adjustments were made three-dimensionally in the YCbCr color space along the black-white, red-green, and blue-yellow axes, chosen for its ease of navigation compared to intensity-based spaces such as RGB. Observers completed adjustments for both left and right test fields shown in Figure 2a. During trials, observers could switch between test fields without a time limit to adjust colors. Note that this task relies on each observer's internal criterion for what is considered white, unlike asymmetric matching, which uses an external reference (Arend & Reeves, 1986). Consequently, we analyzed the degree of color constancy relative to each observer's setting under white skylight conditions, assuming these settings reflect their internal criteria on what is white.

Observers viewed the scene binocularly. Before starting the adjustment for each illuminant condition, observers adapted to the scene for 60 s. The initial chromaticity and luminance of the test field were randomly selected for each trial. Although the order of experimental conditions was randomized, observers could detect changes in illuminant color. More detailed experimental conditions are described in the following sections.

## Experiment 1

### Experimental Condition

**Figure 4.**
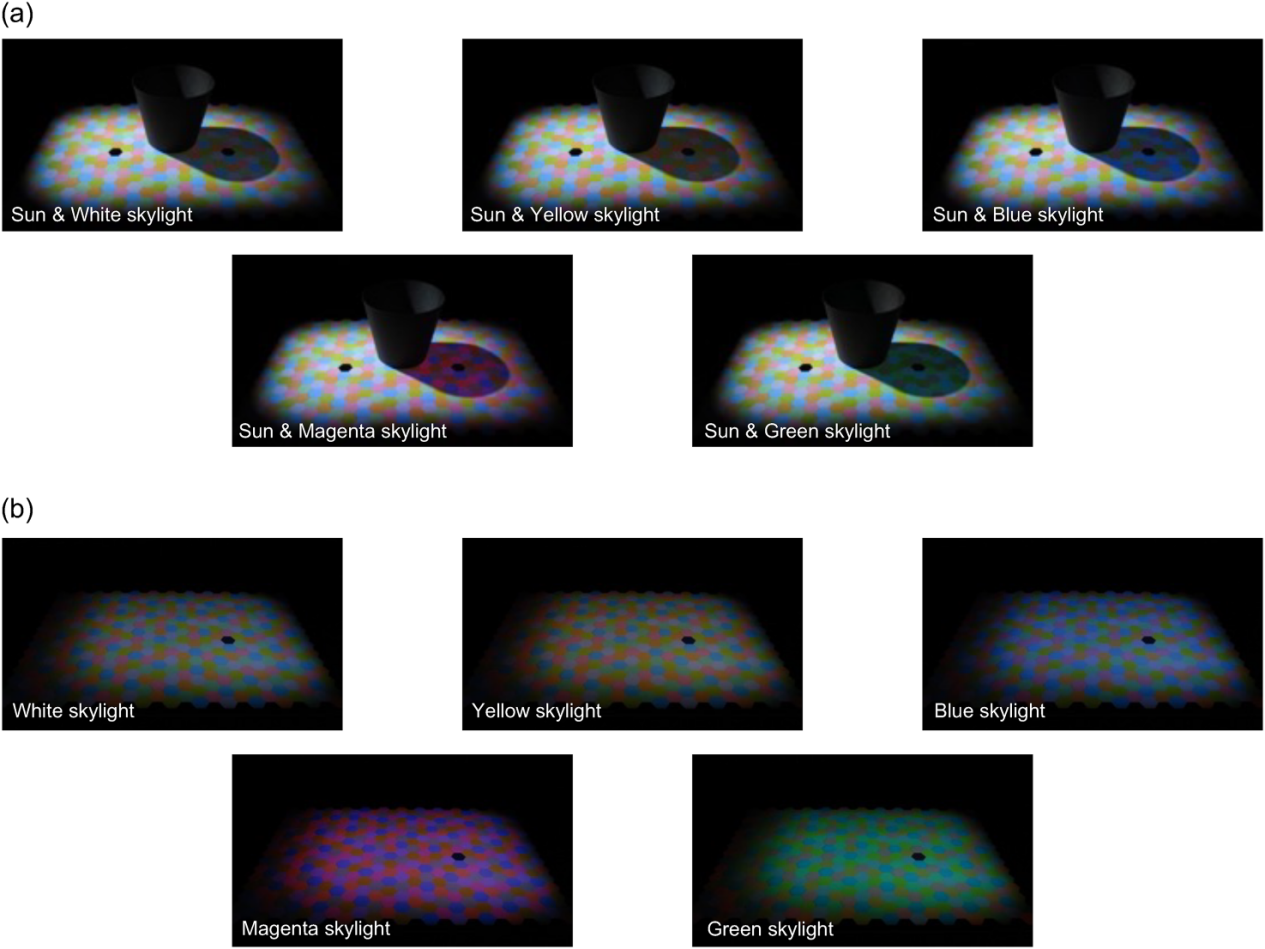
Experimental scenes from Experiment 1, captured approximately from the observers’ viewpoint. (a) Main condition (sunlight and skylight condition) where the scene was illuminated by two independent light sources, with a black object placed centrally to create a shadow. (b) Skylight condition (control) lit by the right projector only, representing a single-illuminant scene where baseline color constancy was measured. In both panels, the black hexagon region(s) correspond to the test field(s).

Figure 4 illustrates the experimental scenes used in Experiment 1, captured from approximately the observers’ viewpoints. Each image displays variations in skylight colors: white, yellow, blue, magenta, and green. Panel a depicts the “sunlight & skylight condition” with both left and right projectors on. Panel b shows the “skylight condition,” where the scene was illuminated only by the skylight from the right projector. This served as a control condition because the scene was lit by a single illumination, similar to traditional color constancy experiments. Each block consisted of five skylight color conditions, and each session included two blocks (sunlight & skylight and skylight-only conditions). All observers completed a total of seven sessions.

### Results

**Figure 5.**
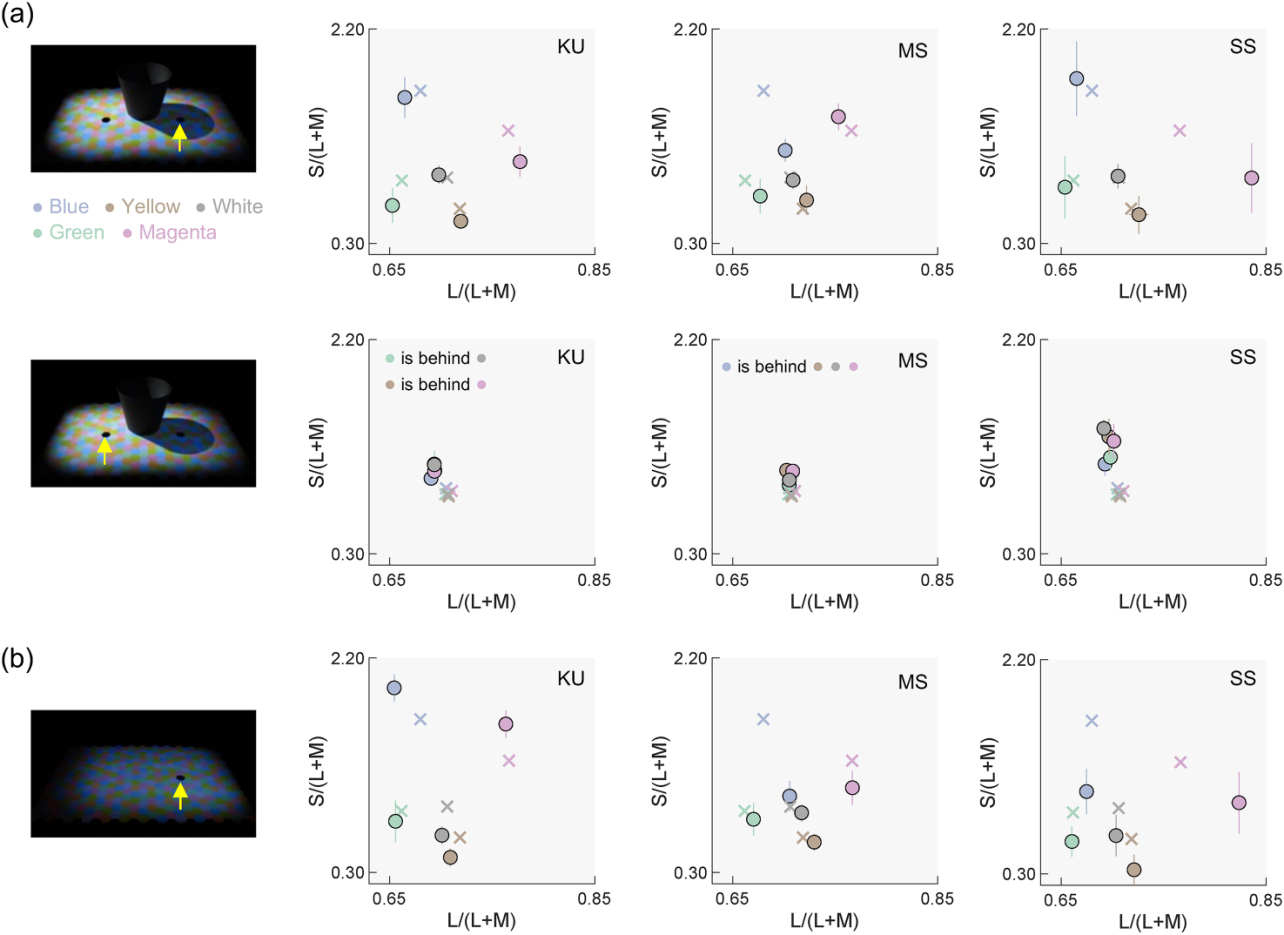
(a) Observer settings for three observers (KU, MS, and SS) for the right and the left test fields (as depicted by the yellow arrow in each image) under the sunlight and skylight condition. Each data point represents the average of seven settings. Circles indicate the observer settings, while crosses denote the chromaticities of the test illuminants measured at the position of the test field. Error bars show the standard error across settings. (b) Observer settings for the skylight-only condition.

Figure 5 presents achromatic settings made by each observer (KU, MS, and SS) for both the sunlight and skylight condition (Panel a) and the skylight condition (Panel b). The yellow arrow in the images shows the side of the test field. Circles represent observer settings, while crosses depict chromaticities of the test illuminants. For the right test field, observer settings tended to shift towards the chromaticity of the skylights compared to settings under the white skylight condition, demonstrating varying degrees of color constancy. For the left test field, achromatic adjustments clustered around the chromaticity of sunlight as expected, irrespective of skylight color, since the skylight was much weaker than the sunlight and had minimal effect on the left test field.

**Figure 6.**
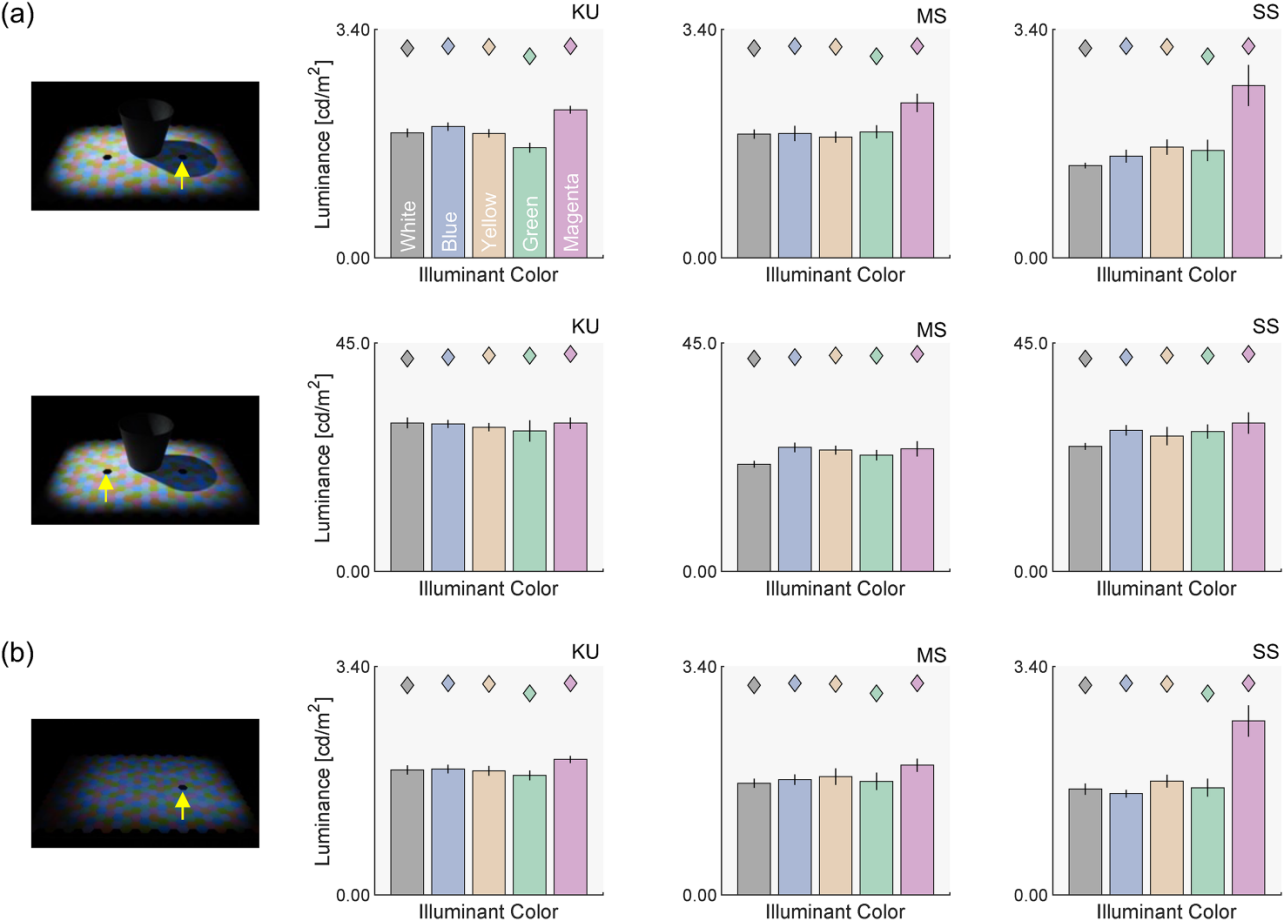
(a) Observers’ luminance settings under the sunlight and skylight condition. Each bar represents the average of seven settings. Diamonds depict the luminance of the test illuminants. Error bars show the standard error across settings. (b) Luminance settings for the skylight-only condition.

Figure 6 presents luminance settings for each observer. In the sunlight and skylight condition (Panel a), all observers show a consistent trend, with minimal differences across skylight colors although slightly higher values are shown for the magenta condition in MS and SS. This consistency is expected, as the luminances of the test illuminants (represented by colored diamonds) were approximately equated across skylight colors. The pattern of settings is similar for both the left test field under the sunlight and the right test field within the cast shadow, indicating that observers compensate for the large intensity difference between nonshadowed and shadowed regions. The results closely align with those in the skylight-only condition (Panel b). Notably, all bars fall substantially below the theoretical limit for a surface (colored diamonds), meaning the white surfaces observers produced were significantly darker than an actual white surface with 100% reflectance across wavelengths. This finding is consistent with previous color constancy studies using the same task (Morimoto et al., 2016, 2021a).

**Figure 7.**
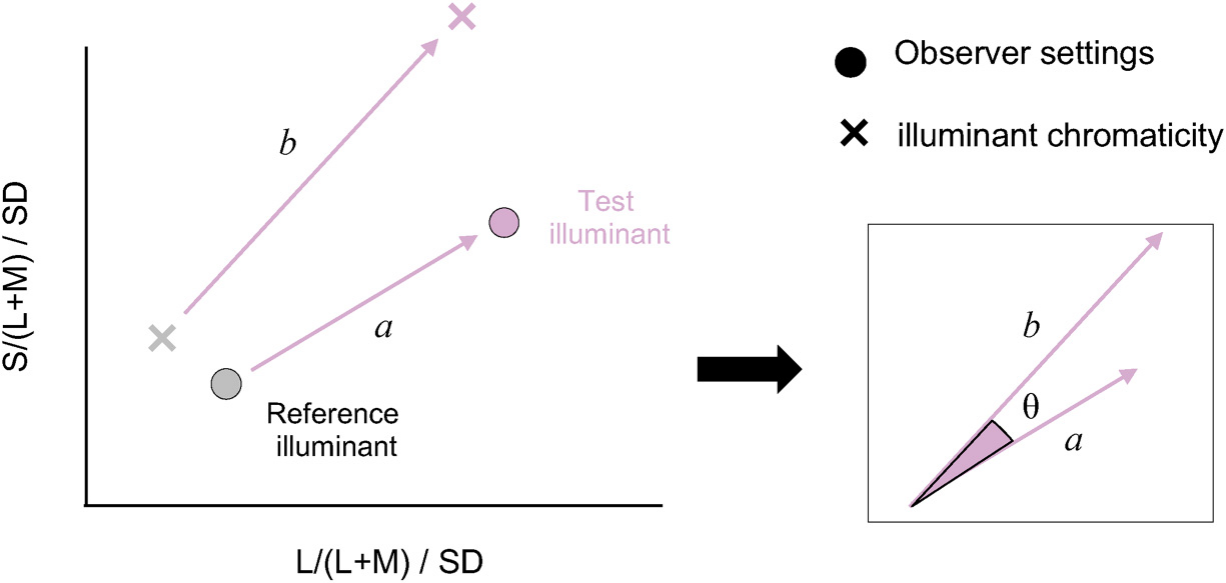
Method for computing a constancy index. Here, *b* denotes the distance between chromaticities of the test illuminant (blue, yellow, green, or magenta) and the reference illuminant (white illuminant) and *a* denotes the distance of achromatic settings. The constancy index is defined as *a*cos*θ*/*b*.

Next, as illustrated in Figure 7, the degree of color constancy was quantified using the distance between the chromaticity of the illuminants (defined as b) and the distance between the chromaticity of the achromatic settings made by observers (defined as a). The reference illuminant was the white skylight. Since the MacLeod-Boynton chromaticity diagram is not a perceptually uniform color space, we adjusted the scale along the horizontal and vertical axes by dividing each axis by the standard deviation of observer settings for the white skylight condition, separately for each observer. The constancy index (*CI*) was defined as shown in Equation (1). This definition is equivalent to a Brunswick ratio that incorporates the vector angle between perceptual and physical illuminant shifts (Foster, 2011).

(1) CI=acosθ/b.

**Figure 8.**
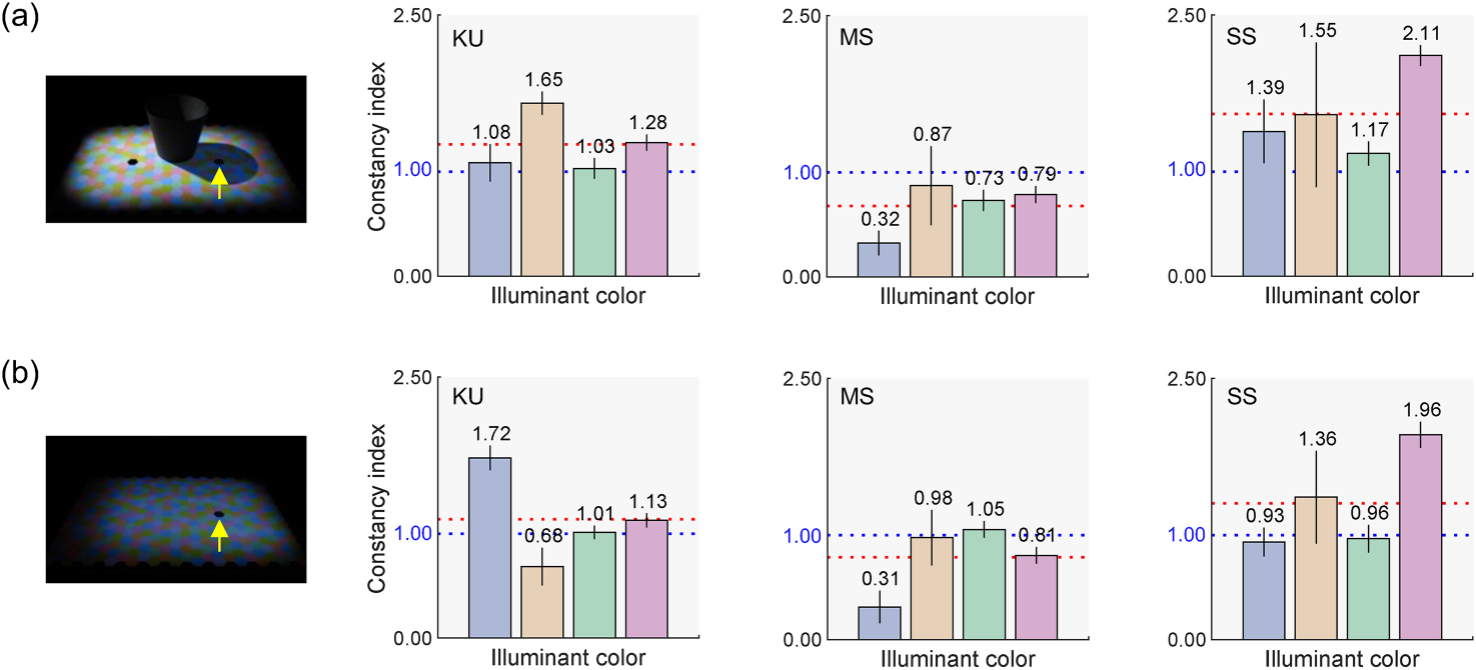
(a) Constancy indices (CIs) for each observer under the sunlight and skylight condition. (b) CIs for the three observers under the skylight-only condition. The error bars indicate ± 1.0 S.E. In both Panels (a) and (b), the blue dotted line represents the perfect constancy, while the red dotted line shows the average across the four skylight colors.

A value of zero indicates no color constancy, a value of one represents perfect constancy, and a value greater than one indicates over-correction of the illuminant color. Figure 8a and b shows the CIs for all three observers under the sunlight and skylight condition and the skylight-only condition, respectively. There are individual differences. For KU, the CI for the blue skylight condition is lower under the sunlight and skylight condition than under the skylight-only condition, while for the yellow skylight condition, the trend is the opposite. For green and magenta skylights, CIs were close to 1.0 for both conditions. For MS, the CI was lower for the blue skylight condition than for the other skylight colors, but the overall trend was similar between the two conditions. For SS, the trend was similar between the conditions, although CIs differed across skylight colors. Importantly, the overall patterns of CIs between the two conditions were roughly similar for all observers, except for the blue and yellow skylight conditions for KU. When averaged over the four skylight conditions and the three observers, the CI was 1.16 for the sunlight and skylight condition, and 1.08 for the skylight-only condition, two-tailed *t*-test, *t*(2) = 0.847, *p* = .486. This suggests that human color constancy within cast shadows (sunlight and skylight condition) is as effective as in a single illuminant condition (skylight-only condition), demonstrating the visual system's ability to handle multiple illuminant conditions. The varying effects of skylight color depending on the observer suggest that humans may not consistently rely on prior knowledge about the specific color of illuminants reaching cast shadows in natural outdoor environments. However, it is also possible that the color distribution of surfaces within the cast shadows provided sufficient cues about the illumination color, reducing the need to rely on prior knowledge of typical color of illumination, which may explain the lack of effect.

## Experiment 2

The primary purpose of Experiment 2 was to examine whether recognizing a shadow as a shadow is crucial for color constancy within the cast shadow. To test this, we masked the penumbra of the cast shadow with black hexagons (Hering, 1964). Additionally, since most observers were naive to the experiment's purpose, we provided clear instructions on the criterion for the achromatic setting task. Otherwise, the experimental procedure and task were identical to those in Experiment 1.

### Instruction for the Experimental Task and the Criterion

Nine out of twelve observers in this experiment were naive to its purpose and had no specialized knowledge about human color vision. We anticipated that they might find it challenging to complete the task without careful instructions. Matching criteria are known to significantly influence the degree of color constancy. A previous study (Arend & Reeves, 1986) demonstrated that performing asymmetric matching based on a paper-match criterion resulted in a substantially higher degree of color constancy compared to matching based on color appearance (“hue-and-saturation” match). Although our task did not require direct matching, the same concern applies. For additional discussion, see studies (Radonjic & Brainard, 2016; Reeves et al., 2008).

**Figure 9.**
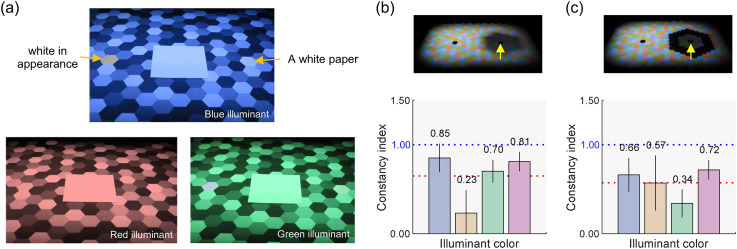
(a) Practice session to help observers distinguish between two matching criteria for achromatic adjustments. (b) Constancy indices for the no-outline condition. (c) Constancy indices for the outlined condition. The bars represent the mean constancy indices across 12 observers. For both panels, the error bars indicate ± 1.0 S.E. across the observers, and the blue and red dotted lines represent the perfect constancy and the average across four skylight colors, respectively.

To help observers understand the criteria, we set up a few practice trials as shown in Figure 9a. The sheet used contained only achromatic colors and was not used in the main experiment. We placed a large white paper at the center of the scene to demonstrate how a real white paper would appear when the illuminant color changed. The scene was lit using a single illumination generated by activating only one of the R, G, or B phosphors of the right projector. This practice scene did not contain any cast shadow.

Observers were then asked to adjust the chromaticity and luminance of the left and right test fields using different criteria. For the right test field, observers adjusted the chromaticity and luminance to make it appear as a full white surface under the test illumination (the criterion used in the actual experiment). For the left test field, they adjusted it to simply appear white. Once an experimenter confirmed that the observer could differentiate between the criteria by comparing the matching results between the test fields, the practice session ended and the main experiment began. Although observers did not receive feedback on their settings, the examples provided were sufficient for them to understand the criterion difference.

### Experimental Condition

As shown in Figure 2b, the scene configuration used in Experiment 2 is similar to that of Experiment 1, but we removed the black object at the center and inserted a paper (“a paper to create a cast shadow”) to block light from the left projector, casting a shadow onto the scene. Observers were unaware of this paper's presence. If instead we had kept the black object, observers would perceive the darkened region as a shadow regardless of our experimental manipulation. In the “no-outline” condition (Figure 9b), we expected the shadow to appear as a shadow. In contrast, in the “outlined” condition, we anticipated that the perception of the shadow would weaken as the penumbra was masked by black hexagons (Figure 9c). Each block had five skylight color conditions, and each session consisted of two blocks (no-outline and outlined conditions). All observers completed two sessions.

### Results

Figure 9b and c reports CIs in Experiment 2. To compute these indices, due to the limited number of trials per observer, we divided the *L*/(*L* + *M*) and *S*/(*L* + *M*) axes by the standard deviation of all observers’ settings for the white skylight condition, using a common scaling value for all observers. We found overall lower constancy indices than those in Experiment 1. The influence of skylight color is not apparent, nor is the effect of outlining the cast shadow. A key difference between Experiments 1 and 2 was the presence of an obvious cue to the cast shadow (i.e., the cup-shaped black object), which might have contributed to the higher degree of constancy in Experiment 1. To investigate this, we compared CIs for KU and MS, who participated in both experiments. For KU, CIs were 1.26 in Experiment 1 (sunlight and skylight condition) and 0.85 in Experiment 2 (no-outline condition) when averaged across skylight colors. For MS, the trend was reversed, with CIs of 0.68 in Experiment 1 and 0.89 in Experiment 2. Thus, the effect of the obvious cue is not evident.

A two-way repeated-measures ANOVA was conducted with four skylight colors (blue, yellow, green, and magenta) and two outline conditions (no-outline and outlined) as within-subject factors for the *CIs*. The main effects of the skylight color and outline condition were not significant, *F*(3, 33) = 1.84, *p* = .159, *F*(1, 11) = 0.26, *p* = .620, respectively. The interaction between the two factors was also not significant, *F*(3, 33) = 1.34, *p* = .278.

These results confirmed that even with a relatively large number of observers, color constancy was maintained to some extent within cast shadows, though the degree decreased compared to Experiment 1. There was no effect from outlining the edge of the shadow, suggesting that observers were still able to infer the influence of the illuminant on the cast shadow. Additionally, there was no effect of skylight color, again indicating little evidence for the use of prior knowledge about skylight color. The reduction of color constancy could simply be attributed to the higher number of non-naïve participants in Experiment 2. To test this, we conducted a Welch's *t*-test on the constancy indices, averaging across four skylight colors for three non-naïve participants and nine naïve participants. The results indicated no significant difference in constancy indices between the two groups, *t*(8) = 2.30, *p* > .05. Therefore, the reduction in constancy in Experiment 2 is unlikely to be solely due to a higher number of naïve participants. However, we acknowledge the limitation of this statistical test due to the imbalance in group sizes.

## General Discussion

It is a mathematically ill-posed problem to accurately judge whether a material is bright white in a blue shadow or dark blue under white illumination. We encounter this challenge in everyday life, yet there has been little experimental focus on color constancy within cast shadows. To address this, we conducted two psychophysical experiments. Results from Experiment 1 confirmed that color constancy in shadows is as effective as in a single-illuminant scene. Our experimental manipulations, including changes to skylight color and outlining the shadow's edge, did not significantly affect the degree of color constancy. Each point will be further discussed below.

One interesting observation was that the color of skylight had little systematic effect on adjustment results. A visual strategy relying on a prior about the typical color of skylight would predict observer settings in the bluish or yellowish regions of the color space. This expectation may be reasonable because shadowed regions in natural environments exhibit chromatic regularities. On a sunny day, the color temperature of skylight varies between about 8,000 and 25,000 K, with variation tightly restricted along the CIE daylight locus (Morimoto et al., 2022). However, contrary to this, observer settings were better predicted by the skylight color used in the experiment, aligning with controversies about the existence of a daylight prior in human color constancy (e.g., Delahunt & Brainard, 2004; Hurlbert, 2019). A prior may only have an effect under conditions where there is uncertainty about illumination. In both experiments, we used six differently colored hexagons, which might have provided sufficient cues to infer the illuminant color, reducing reliance on prior knowledge. To test this, we measured the degree of color constancy for 12 observers using uniform grey papers instead of the hexagons, as in Experiment 2. The results showed that color constancy dropped to nearly zero for all skylight colors, including blue. This supports the view that observers do not use specific prior knowledge when judging illuminant color within cast shadows. Additionally, it is worth noting that cast shadows occur in indoor environments, where the illuminant color is not limited to daylight colors. This might explain why skylight color did not show systematic effects in this study.

An influential work by Helson (1947) on adaptation-level theory, describes how perception is determined relative to an adaptation level shaped by the surrounding stimuli. In the context of color constancy, setting a reference point—such as a white point in a scene—helps normalize color perception by accounting for the prevailing illumination, providing a basis for perceptual constancy. Similarly, in computer vision applications, color constancy is often approached as the challenge of identifying a single suitable white point within a scene. This point is then used to adjust the colors throughout the scene, effectively minimizing the impact of illumination. However, in complex scenes with cast shadows, a single reference may not suffice, necessitating multiple local adaptation points. Consequently, a mechanism that calculates scene statistics globally (Buchsbaum, 1980; Golz & MacLeod 2002) may not adequately explain the color constancy observed in this experiment. Instead, it may be essential to segment scenes into smaller fragments (Golz, 2008; Gilchrist et al., 1999), or perform such calculations on a per-object basis (Rodríguez et al., 2024). The original version of von Kries adaptation (von Kries, 1905) proposes adjusting cone sensitivity spatially locally rather than globally. This enables distinct normalization in shadowed and sunlit areas and may serve as a potential mechanism for establishing multiple anchors across the visual field. A key aspect of our experiments was that the local illumination conditions near the right test field were consistent between the sunlight and skylight condition and the skylight-only condition. Therefore, mechanisms that consider the immediate surroundings of the test field to infer the illuminant color should align with the pattern of constancy indices observed in this study.

Given these considerations, detecting a cast shadow in the visual field appears to be beneficial for determining how a scene should be segmented into smaller fragments. This is a primary reason we hypothesized that the spatial structure of the shadow serves as a cue for identifying a cast shadow. However, outlining the shadow had minimal impact on color constancy in this study. We propose several potential explanations for this. First, some observers may have continued to perceive the outlined shadow as a shadow, even after the spatial intensity regularity was disrupted. Second, the shadowed area might have seemed illuminated by a spotlight (Khang & Zaidi, 2004). Alternatively, observers may have relied solely on color and luminance differences to detect the shadow rather than boundary information (Kingdom et al., 2004). In either scenario, observers could maintain a distinct reference for the cast shadow and other regions in the scene, thereby preserving color constancy.

In Experiment 2, we observed a moderate degree of color constancy among mostly naïve observers. We suspected that two factors may have influenced these results. The first significant factor was the explicit instructions we provided regarding adjustment criteria, which have been reported in previous studies (Radonjic & Brainard, 2016; Reeves et al., 2008). The second factor was the observers’ awareness that the illuminant color was being changed during the experiment. To investigate these factors, we conducted a follow-up experiment identical to Experiment 2, but without the practice trials shown in Figure 9a, and we instructed observers to leave the experimental room when the illuminant color was changed. As a result, we found that the constancy indices dropped to nearly zero. While it remains unclear which factor contributed to this outcome, or to what extent, since both factors were manipulated simultaneously, these findings demonstrate that instructions and/or experimental procedures significantly affect color constancy in shadows. This is consistent with reports from traditional color constancy studies in single-illuminant environments (Radonjic & Brainard, 2016).

Color constancy has long been a central focus in color vision research, with its mechanisms explored through various experimental manipulations. However, the complexities of real-world illuminant conditions suggest that our knowledge of color perception in practical situations may still be limited. The current investigation into the effects of cast shadows has the potential to enhance our understanding of color constancy.
